# A Time-Resolved In Situ SAXS Method for Real-Time Monitoring of Lipid Nanoparticles Assembly

**DOI:** 10.3390/membranes16060192

**Published:** 2026-06-02

**Authors:** Ke-Meng Li, Panqi Song, Xiao-Peng He, Na Li

**Affiliations:** 1 Key Laboratory for Advanced Materials and Joint International Research Laboratory of Precision Chemistry and Molecular Engineering, Feringa Nobel Prize Scientist Joint Research Center, School of Chemistry and Molecular Engineering, East China University of Science and Technology, 130 Meilong Road, Shanghai 200237, China; likemeng2024@sari.ac.cn; 2 National Facility for Protein Science Shanghai, Shanghai Advanced Research Institute, Chinese Academy of Sciences, Shanghai 201210, China; songpq@sari.ac.cn

**Keywords:** time-resolved small-angle X-ray scattering, lipid nanoparticles, self-assembly, nanostructure

## Abstract

Lipid nanoparticles (LNPs) have emerged as popular nucleic acid delivery systems, yet the dynamic mechanisms related to their self-assembly and structural maturation remain insufficiently understood due to the limitations of traditional offline characterization tools. This study establishes a time-resolved (TR) in situ small-angle X-ray scattering (SAXS) methodology to monitor the structural evolution of LNPs during microfluidic formulation and subsequent maturation. By integrating a dual-channel microfluidic mixing system with a SAXS measurement platform, we successfully captured the real-time scattering profiles of both empty and messenger RNA-loaded nanoparticles (mRNA-LNPs). The results demonstrate distinct assembly pathways for empty-LNPs and those encapsulated with mRNA. The empty-LNPs undergo a gradual transition toward periodic nanostructures, whereas mRNA-LNPs exhibit rapid complexation into stable subunits followed by hierarchical assembly. Furthermore, the platform effectively tracked nanoscale structural rearrangements during a microfluidic dilution process, revealed by subtle shifts in scattering peaks and internal periodicity. Overall, this time-resolved approach provides a robust experimental framework for capturing transient intermediate states, offering a valuable tool to elucidate molecular assembly mechanisms and facilitate the rational design of next-generation nanomedicines.

## 1. Introduction

Lipid nanoparticles (LNPs) have emerged as the prevailing system for the delivery of nucleic acid therapeutics [1,2], most notably highlighted by the global success of mRNA-based COVID-19 vaccines [3,4]. The biological performance of LNPs, including their endosomal escape capability and transfection efficiency, is profoundly dictated by their internal nanostructure [5,6,7,8,9]. Research has demonstrated that different lipid combinations tend to form various structures, such as lamellar phases, inverse hexagonal phases, or cubic phases [10,11], and that specific lipid arrangements directly correlate with the potency and safety profiles of the delivery system. It has been demonstrated in the study by Yu et al. [12] that the evolution of lyotropic liquid crystalline mesophases, such as inverse cubic and hexagonal phases, correlates strongly with mRNA transfection potency during endosomal acidification. Pattipeiluhu et al. [13] have also pointed out that liquid crystalline inverse hexagonal phases in LNPs enhance silencing efficiency. Consequently, achieving a precise understanding of these “structure–function” relationships is vital for the rational design of next-generation LNPs.

Despite the importance, the characterization of LNPs nanostructures faces significant technical bottlenecks. Currently, most characterization tools rely on “offline” or ex situ techniques, such as Dynamic Light Scattering (DLS) and Cryogenic Electron Microscopy (Cryo-EM) [14,15]. Leung et al. and Kulkarni et al. [16,17] combined cryo-EM with molecular dynamics simulations to provide deep insights into the dynamic assembly pathways and structural characteristics of LNPs. Furthermore, Small-Angle Neutron Scattering (SANS) has been utilized to resolve the internal organization of LNPs by leveraging contrast variation between different components. Davies et al. [18] employed SANS to investigate the spatial distribution of internal components, providing strong evidence that the rofleponide-C14 prodrug is not homogeneously distributed within the MC3-LNPs, but rather is preferentially located in the outer shell region. While these tools provide high-resolution snapshots of the final product, they are fundamentally limited by “sampling bias” and a “time-lag” effect [19]. The self-assembly of LNPs is a highly dynamic process occurring on the second-to-millisecond timescale [20]. Traditional endpoint measurements fail to capture the transient intermediate states and the kinetic pathways through which individual lipid components organize into complex nanoparticles [21,22].

Small-angle X-ray scattering (SAXS) offers a powerful solution for resolving these challenges. As a non-invasive technique based on the principle of elastic X-ray scattering, SAXS can provide precise information regarding particle shape, internal periodicity (d-spacing), and electron density distributions at the nanometer scale [23,24,25,26]. Padilla et al. [27] employed SAXS in combination with other high-resolution biophysical methods, such as analytical ultracentrifugation and field-flow fractionation, to accurately quantify the intrinsic polydispersity in size, shape, and cargo loading of mRNA-LNPs, thereby establishing critical structure–function relationships. Xin et al. [28] developed multicompartment LNPs (A3-DM/DL-LNPs) utilizing a novel “M-type” ionizable lipid with a unique polar headgroup composed of piperazine and di-imidazole. Through SAXS analysis, they demonstrated that this specific molecular configuration breaks the constraints of traditional inverse micellar or lamellar structures, inducing the formation of non-lamellar, sponge-like multicompartment networks with high membrane heterogeneity and wide d-spacing, which significantly enhances endocytosis and endosomal escape efficiency. While SAXS has been widely utilized to characterize the equilibrium states of various lipid-based drug delivery systems [29,30,31,32,33], its potential for real-time, in situ monitoring during the formulation and maturation stages remains underexplored. By integrating SAXS with microfluidic devices, real-time, in situ, time-resolved SAXS (TR-SAXS) presents a promising frontier for tracking structural evolution in a continuous-flow environment.

In this work, we developed a methodology for the in situ observation of nanostructural transitions during LNP formulation. By coupling a microfluidic mixing device with the synchrotron SAXS facility, we collected the real-time scattering profiles of LNPs throughout both the initial assembly and subsequent maturation processes. The reliability of the setup was first validated by comparing the SAXS profiles of empty LNPs and mRNA-LNPs prepared by both offline and online methods, which demonstrated high comparability between the two methods. The feasibility of this approach was further demonstrated by monitoring the subtle evolution of scattering profiles, which reflect the gradual structural rearrangements occurring during the lipid assembly process. Our study establishes a robust methodological framework for tracking the dynamic nanostructural evolution of LNPs, offering a practical tool for comparative formulation screening and the optimization of microfluidic manufacturing parameters.

## 2. Materials and Methods

### 2.1. Materials

DLin-MC3-DMA (MC3) was purchased from Shanghai SCR-Biotech Co., Ltd. (Shanghai, China), 1,2-dierucoyl-sn-glycero-3-phosphocholine (DSPC), and 1,2-dimyristoyl-rac-glycero-3-methoxypolyethylene glycol-2000 (DMG-PEG2000) were purchased from Avanti^®^ Polar Lipids (Alabaster, AL, USA). Cholesterol was purchased from Sigma Aldrich (Shanghai, China), and GFP-mRNA was purchased from Perfect mRNA Biotechnology (Hangzhou, China). The customized micromixer chip features a complex channel geometry with a channel width of 0.2–0.4 mm and a depth of 0.1–0.3 mm, specifically designed to enhance mixing efficiency by integrating chaotic advection into the laminar flow streams.

### 2.2. LNPs Formulation

LNPs were formulated using a microfluidic mixing device. The organic phase was prepared by dissolving MC3, DSPC, cholesterol, and DMG-PEG2000 in ethanol at a molar ratio of 50:10:38.5:1.5, reaching a final total lipid concentration of 10 mM. For the preparation of empty LNPs, a sterile citrate buffer (0.1 M, pH 4.0) was employed as the aqueous phase. For mRNA-loaded LNPs (mRNA-LNPs), GFP-mRNA was diluted in the same citrate buffer to a concentration of 0.09 mg·mL^−1^, maintaining an N/P ratio of 6. The organic and aqueous phases were loaded into separate syringes and infused into a microfluidic chip (Chemyx Fusion 200X Syringe Pump, New York, NY, USA) at a flow-rate ratio (FRR) of 3:1 (aqueous-to-organic) and a total flow rate (TFR) of 3.6 mL/min.

### 2.3. Batch Mode SAXS Measurement

SAXS measurement was performed at the BL19U2 beamline of the National Facility for Protein Science Shanghai (NFPS), located at the Shanghai Synchrotron Radiation Facility (SSRF). The incident X-Ray beam was operated at 12 keV, corresponding to a wavelength (λ) of 0.103 nm. Two-dimensional scattering patterns were collected using a PILATUS3X 2M detector (Dectris, Baden-Daettwil, Switzerland). The sample-to-detector distance was set to 2668 mm, enabling detection of the scattering vector *q*, defined as *q* = 4πsin *θ*/*λ*, where *θ* represents half of the scattering angle, over a range of 0.0065–0.4250 Å^−1^. A flow cell made of a quartz capillary with an inner diameter of 1.5 mm was used to sample an aliquot of the LNPs solution. The exposure time was fixed at 1 s per scattering pattern, and 20 consecutive scattering patterns were collected and compared for data averaging. Preliminary data reduction and processing were performed using the BioXTAS RAW software (version 2.2.1) package [23].

### 2.4. Experimental Setup for In Situ TR-SAXS

The in situ TR-SAXS platform was established at the BL19U2 beamline. A dual-channel microfluidic syringe pump system was positioned inside the experimental hutch and operated remotely. Syringes fixed on Pump 1 and Pump 2 were connected to the two inlets of a microfluidic chip, respectively. To enable real-time data collection without radiation damage to the microfluidic chip, the outlet of the chip was connected to a quartz capillary (inner diameter: 1.5 m; wall thickness: 10 μm). The SAXS patterns of the flowing liquid within the capillary were collected continuously during the processes.

### 2.5. Monitoring of Online LNPs Formulation

To observe the structural evolution during LNP formulation, a dual-channel microfluidic syringe pump system was installed within the experimental hutch and operated remotely. The detailed experimental setup is as described in Section 2.4. Syringes containing the organic phase (lipid mixture) and the aqueous phase (citrate buffer or mRNA solution) were loaded onto Pump 1 and Pump 2 for the preparation of empty LNPs and mRNA-LNPs, respectively. Initially, Pump 2 was started to fill the quartz capillary with the lipid solution at a flow rate of 0.9 mL/min. Subsequently, the aqueous phase was injected by starting Pump 1 at a flow rate of 2.7 mL/min. Synchronously with the activation of Pump 1, SAXS profiles of the flowing solution within the capillary were then recorded continuously throughout the assembly and maturation process of LNPs. The exposure time was fixed at 1 s per frame, and 70 consecutive scattering patterns were collected in general.

### 2.6. Monitoring of Online LNPs Dilution

To investigate the structural changes during the dialysis stage, here we employed an online microfluidic dilution procedure to simulate the pH shift during the real dialysis process. Raw empty LNPs and mRNA-LNPs were first prepared offline according to the procedure mentioned in Section 2.2. For the online dilution experiments, PBS (pH 7.4) and the raw LNP solutions were loaded into Pump 1 and Pump 2, respectively. Both pumps were started simultaneously to initiate the mixing process. Concurrent with the pump activation, SAXS data collection was initiated with an exposure time of 1 s per pattern, and a total of 50 consecutive patterns were recorded for each sample.

## 3. Results and Discussion

### 3.1. Comparability of LNPs Formulated by Offline and Online Methods

**Figure 1:**
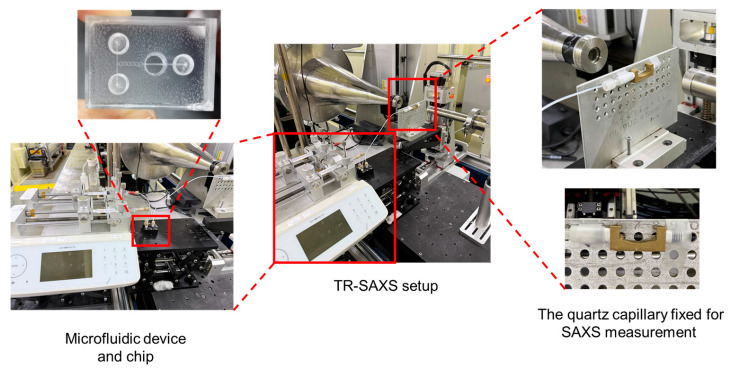
TR-SAXS setup at BL19U2 in SSRF with a dual-channel microfluidic syringe pump system.

To validate the reliability and representativeness of the in situ TR-SAXS setup, we first conducted a comparative study between LNPs formulated by conventional offline methods and those produced by the online microfluidic-coupled system. Initially, empty LNPs and mRNA-LNPs were formulated according to the standard offline protocols described in Section 2.2, and their mesoscopic structures were characterized using SAXS batch mode, as detailed in Section 2.3. Next, with the TR-SAXS experimental setup shown in Figure 1, both empty and mRNA-loaded LNPs were formulated online under identical parameters. The resulting products were collected from the outlet of the quartz capillary and characterized in batch mode as well to ensure consistency in the detection environment.

**Figure 2:**
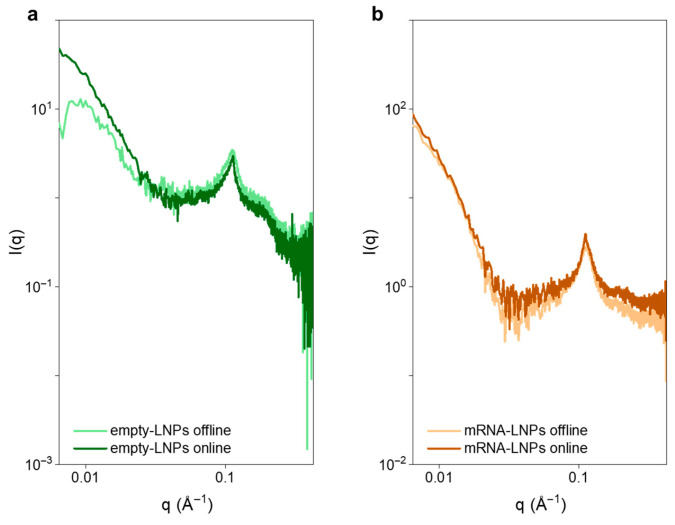
SAXS profiles of (**a**) empty LNPs and (**b**) mRNA-LNPs prepared by offline and online methods.

The SAXS profiles for LNPs prepared with both offline and online methods are shown in Figure 2. For both empty LNPs and mRNA-LNPs, the scattering profiles obtained from the two preparation methods exhibited a high degree of overlap, with almost identical peak positions and consistent scattering features across most of the *q* range. However, while mRNA-LNPs show perfect overlap, the low-*q* intensity of empty LNPs is slightly lower in offline mode than online. This indicates that without mRNA’s structural stabilization, empty LNPs undergo a rapid relaxation from a transient, flow-induced, correlated state to a more stable, equilibrated dispersion within minutes of collection. This structural congruency indicates that the integrated microfluidic mixing and capillary detection system does not alter the fundamental self-assembly or the final nanostructure of the particles.

These results demonstrate that the LNPs formulated and monitored on our TR-SAXS platform are highly comparable to those produced by traditional offline methods, thereby providing a solid experimental foundation and reliable results for the subsequent in situ observation of dynamic assembly and simulated dialysis processes.

### 3.2. In Situ Observation of Structural Evolution During LNPs Formulation

To capture the real-time formation of LNPs, we established an in situ TR-SAXS observation platform at the BL19U2 beamline. As shown in Figure 1, the experimental setup comprises two integrated modules: a microfluidic mixing device and a downstream detection setup. The microfluidic unit, consisting of a dual-channel syringe pump and a specialized chip, was employed for the controlled assembly of both empty LNPs and mRNA-LNPs. This unit was connected to a thin-walled quartz capillary (1.5 mm diameter, 10 μm wall thickness) through a tube, which served as the sample cell for SAXS data acquisition. The detailed operational parameters for the LNPs formulation were consistent with the protocols described in Section 2.5.

**Figure 3:**
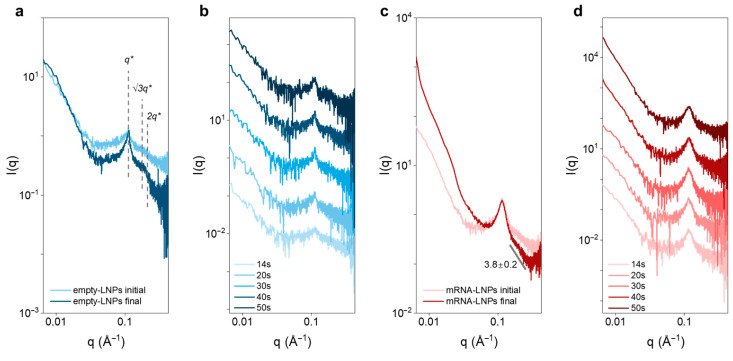
In situ TR-SAXS characterization of LNPs assembly during microfluidic formulation. (**a**) Comparison of SAXS profiles for empty LNPs at the initial and final stages of the assembly process. The q* identifies the fundamental peak position, used as a basis for indexing the secondary Bragg peaks. The characteristic Bragg peaks of the *H_II_* phase are indicated by dashed lines with a spacing ratio of $1:\sqrt{3}:2$. (**b**) Time-resolved evolution of SAXS patterns for empty LNPs at representative time points (14, 20, 30, 40, and 50 s). Profiles of 14, 20, 40, and 50 s are multiplied by factors of 0.01, 0.1, 10, and 100, respectively, for better visualization. (**c**) Comparison of SAXS profiles for mRNA-LNPs at the initial and final stages of the assembly process. (**d**) Time-resolved evolution of SAXS patterns for mRNA-LNPs at representative time points (14, 20, 30, 40, and 50 s). Profiles of 14, 20, 40, and 50 s are multiplied by factors of 0.01, 0.1, 10, and 100, respectively, for better visualization.

Using this integrated platform, we first characterized the assembly kinetics of empty LNPs. As shown in Figure 3a, a significant structural transition was observed between the initial mixing stage and the final stabilized state. Specifically, a characteristic scattering peak emerged at *q*~0.1 Å^−1^ toward the end of the assembly process, accompanied by a significant increase in scattering intensity and a narrowing of the peak width. This trend is further illustrated by the time-resolved profiles in Figure 3b, where the progressive sharpening and intensification of the 0.1 Å^−1^ peak from 14 s to 50 s indicate a gradual transition of the lipid mixture from a disordered state (Figure S1) into a well-defined periodic nanostructure. Furthermore, as shown in Figure S2, an enlarged view of the SAXS profiles for empty LNPs at the final formulation stage reveals the presence of a higher-order Bragg peak. These secondary peaks, appearing at scattering vector *q* ratios $1:\sqrt{3}:2$ relative to the primary peak, are consistent with an inverse hexagonal liquid crystalline phase packing within the lipid matrix [25,34]. The clear resolution of these higher-order reflections demonstrates that the established TR-SAXS platform is not only capable of capturing overall particle formation but also provides sufficient structural resolution to probe fine nanostructural transitions and internal lipid organization with a nanometer-scale precision.

Following the validation with empty LNPs, the structural evolution of mRNA-LNPs was monitored in situ. Interestingly, the mRNA-LNPs exhibited a distinct kinetic behavior compared to empty LNPs. As shown in Figure 3c,d, the characteristic peak at *q*~0.1 Å^−1^ showed no significant variations in intensity or shift throughout the observed timeframe. This stability suggests that the negatively charged mRNA molecules and the protonated ionizable lipids (MC3) rapidly associate via electrostatic interactions, forming rather stable assembly subunits within probably a millisecond timescale, which is too rapid to be fully resolved by the current time resolution. Based on the Bragg equation [35] d = 2*π*/*q*, the corresponding characteristic length scale is approximately 5.5 nm, which likely reflects the internal periodicity of the mRNA-lipid complex core [36].

The subsequent morphological maturation of mRNA-LNPs was primarily reflected in the low-*q* region. As shown in Figure 3c,d, a gradual increase in the low-*q* region was accompanied by the emergence of subtle characteristic oscillation features of spherical particles. These results imply a hierarchical assembly mechanism for mRNA-LNPs: the mRNA first rapidly complexes with ionizable lipids to form stable, small structural subunits (length scale ~5.5 nm), followed by integrating with other helper lipid components (DSPC, cholesterol, and PEG lipid) to yield larger, structurally defined nanoparticles. Collectively, these findings demonstrate that the established TR-SAXS methodology is a robust and effective platform for resolving the rapid assembly kinetics and capturing transient intermediate states in LNPs systems, thereby offering valuable insights for the rational design and optimization of diverse LNPs formulations.

It should be noted that the final state of empty LNPs (Figure 3a) and the initial state of mRNA-LNPs (Figure 3c) both exhibited well-defined Guinier regions in the low *q* regime, indicating the compact, monodisperse aggregated non-global structures. Linear fitting of the Guinier plots yielded the values for radius of gyration (*R_g_*) as 13.7~20.4 nm for the final state of empty LNPs and 13.6~16.3 nm for the initial state of mRNA-LNPs, respectively. These dimensions confirmed that both states reach a structurally stable configuration. In contrast, the initial empty LNP state (Figure 3a, light blue curve) exhibits a featureless, gradual decay across the entire low *q* range without a discernible Guinier plateau, indicating that the spatially unconstrained nanoparticles are undergoing free positional fluctuations. This characteristic is consistent with a dispersed, non-aggregated particle distribution prior to the inter-particle association. Moreover, the final-state mRNA-LNPs scattering profile (Figure 3c) displayed a pronounced power-law decay in the high *q* range (0.15–0.30 Å^−1^), with a fitting Porod exponent of −3.8 ± 0.2. The fitting result was in good agreement with the theoretical value of −4 for ideal Porod scattering from smooth, sharp interfaces, and further confirmed that the mature mRNA-LNPs possess well-defined, compact boundaries in overall shape, forming consolidated nanoparticles.

### 3.3. In Situ Monitoring of Structural Rearrangement During LNPs Dilution

Previous studies have demonstrated that environmental pH plays a critical role in regulating the self-assembly and internal organization of LNPs [10,17,37], particularly during the downstream dialysis process, where the solvent shifts from acidic to physiological conditions. To further explore this structural maturation process, we used the TR-SAXS platform to monitor the nanostructural evolution during a simulated dialysis process, achieved via online microfluidic dilution of the raw LNPs.

Initially, raw empty LNPs and mRNA-LNPs were prepared offline, as described in Section 2.2. These raw LNPs were then diluted with PBS (0.01 M, pH 7.4) at different ratios to determine the optimal detection window. As shown in Figure S3, a dilution ratio of 1:5 (raw LNPs:PBS) resulted in insufficient scattering intensity for reliable analysis. Consequently, a moderate dilution ratio of 1:3 was selected for subsequent in situ experiments. Following the description in Section 2.6, the flow rates for Pump 1 (PBS) and Pump 2 (raw LNPs) were set at 2.7 mL/min and 0.9 mL/min, respectively, to maintain this dilution ratio during real-time observation.

**Figure 4:**
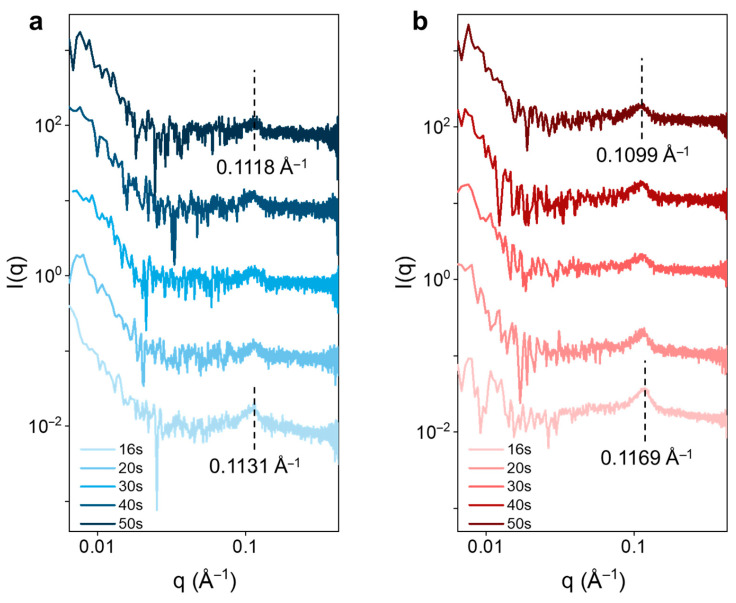
SAXS profiles of (**a**) empty LNPs and (**b**) mRNA-LNPs during the online dilution process at representative time points (16, 20, 30, 40, and 50 s). Profiles of 16, 20, 40, and 50 s are multiplied by factors of 0.01, 0.1, 10, and 100, respectively, for better visualization.

The structural transitions of empty LNPs during the microfluidic dilution are shown in Figure 4a. Upon dilution and the corresponding pH increase, the characteristic scattering peak gradually shifted from *q* = 0.11307 Å^−1^ to 0.11182 Å^−1^. According to the Bragg equation, this corresponds to a subtle increase in the lipid bilayer d-spacing from approximately 5.5 nm to 5.6 nm, indicating a modest increase in characteristic repeat distance and a concurrent reduction in long-range structural ordering. Meanwhile, a slight decrease in peak intensity was also observed, suggesting a relatively subtle reorganization of the internal lipid matrix during the pH transition.

In contrast, the mRNA-LNPs exhibited a more pronounced structural response during the same process (Figure 4b). A significant peak shift was recorded from 0.11693 Å^−1^ to 0.10996 Å^−1^, indicating a substantial expansion of the internal periodic structure from 5.3 nm to 5.7 nm. Additionally, a progressive increase in scattering intensity at the slope of the low-*q* region and the emergence of weak oscillatory features were observed, signifying the growth of the mRNA lipid subunits and the eventual formation of integral, well-defined LNP particles.

Notably, the temporal evolution of mRNA-LNPs is more substantial than that of empty LNPs, highlighting the critical role of mRNA–lipid interactions in driving structural remodeling under physiological conditions. Despite the reduced signal-to-noise ratio at later time points due to dilution, the overall trends remain clearly discernible, demonstrating the capability of the TR-SAXS platform to resolve dynamic structural transitions in complex nanomedicine systems.

## 4. Conclusions

In this work, we established an in situ, time-resolved experimental platform for monitoring nanoscale structural transitions of LNPs using synchrotron SAXS. By integrating a microfluidic mixing system with the SAXS detection platform at BL19U2 beamline located at SSRF, we successfully captured real-time scattering profiles of both empty LNPs and mRNA-LNPs during their primary assembly and subsequent maturation stages.

The established platform facilitated real-time data acquisition with a temporal resolution of 1 s per frame (with the possibility to go down to 3 ms). The reliability of this online approach was validated by the strong agreement between scattering profiles obtained from the online setup and those from conventional offline preparations. The consistency confirmed that the TR-SAXS approach is a representative tool for studying LNP assembly under controlled flow conditions.

Our in situ, real-time observations revealed distinct scattering behaviors for the two LNP systems. Empty LNPs exhibited a gradual emergence of scattering peaks at a *q* ratio of $1:\sqrt{3}:2$, indicative of the transition toward inverse hexagonal phases. In contrast, mRNA-LNPs exhibited the rapid formation of structural features that remained largely stable throughout the observed timeframe, along with a gradual intensity increase in the low-*q* region, reflecting particle growth. Furthermore, the platform effectively captured subtle shifts in scattering peak positions (*d*-spacing) during the microfluidic dilution process, highlighting the sensitivity of LNP nanosctructure to environmental pH variations.

In summary, this work demonstrates that TR-SAXS is a robust and effective methodology for resolving dynamic structural evolutions in LNPs systems. The developed platform provides a practical experimental tool for capturing transient intermediated states during nanomedicine preparation, thereby supporting formulation screening and process optimization. The current design of the microfluidic chip is inherently modular and can be integrated into multi-layered architectures to increase throughput, which is already demonstrated in commercial microfluidic manufacturing systems, such as impingement jets or large-scale microfluidic mixers. The platform can operate under continuous-flow, non-invasive conditions, which aligns with established industrial processes, such as Continuous Manufacturing (CM) and Process Analytical Technology (PAT) frameworks widely adopted in the pharmaceutical and materials industry. It can be implemented using laboratory-scale or synchrotron-based SAXS to monitor the structural integrity and batch-to-batch consistency of LNPs during the scale-up process, providing a molecular-level feedback loop to optimize critical process parameters (CPPs) such as flow rate ratios or mixing temperatures. We also note ongoing advances in advanced high-brilliance laboratory SAXS instruments and automated microfluidic handling systems that will further bridge the gap between academic prototyping and industrial implementation. Future work may integrate this platform with complementary characterization techniques to achieve a more comprehensive understanding of the LNP self-assembly mechanisms.

## Data Availability

The original contributions presented in this study are included in the article/Supplementary Material. Further inquiries can be directed to the corresponding authors.

## Supplementary Materials

The following supporting information can be downloaded at https://www.mdpi.com/article/10.3390/membranes16060192/s1, Figure S1: SAXS profiles of lipid mixture; Figure S2. Enlarged view of SAXS profiles of empty LNPs formulated online at the final stage; Figure S3: SAXS profiles of (a) empty LNPs and (b) mRNA-LNPs diluted offline at different ratios. Profiles of 1:5, 1:4, 1:2, 1:1 and raw LNPs are multiplied by a factor of 0.01, 0.1, 10, 100, and 1000, respectively, for better visualization.
