# Supplementary material for: A Time-Resolved In Situ SAXS Method for Real-Time Monitoring of Lipid Nanoparticles Assembly
Source: Membranes (Basel). 2026 Jun 2;16(6):192. doi: 10.3390/membranes16060192 (PMC13304025; doi:10.3390/membranes16060192)
Supplement: Supplementary file 1 [file membranes-16-00192-s001.zip › membranes-4266652-supplementary.pdf]

membranes

Supplementary materials

A Time-Resolved In Situ SAXS Method for Real-Time Monitoring of Lipid Nanoparticles Assembly

Ke-Meng Li [1,2], Panqi Song [2], Xiao-Peng He [1,*] and Na Li [2,*]

[1] Key Laboratory for Advanced Materials and Joint International Research Laboratory of Precision Chemistry and Molecular Engineering, Feringa Nobel Prize Scientist Joint Research Center, School of Chemistry and Molecular Engineering, East China University of Science and Technology, 130 Meilong Rd, Shanghai 200237, China; likemeng2024@sari.ac.cn

[2] National Facility for Protein Science Shanghai, Shanghai Advanced Research Institute, Chinese Academy of Sciences, Shanghai 201210, China; songpq@sari.ac.cn

* Correspondence: xphe@ecust.edu.cn (X.-P.H.); lina02@sari.ac.cn (N.L.)

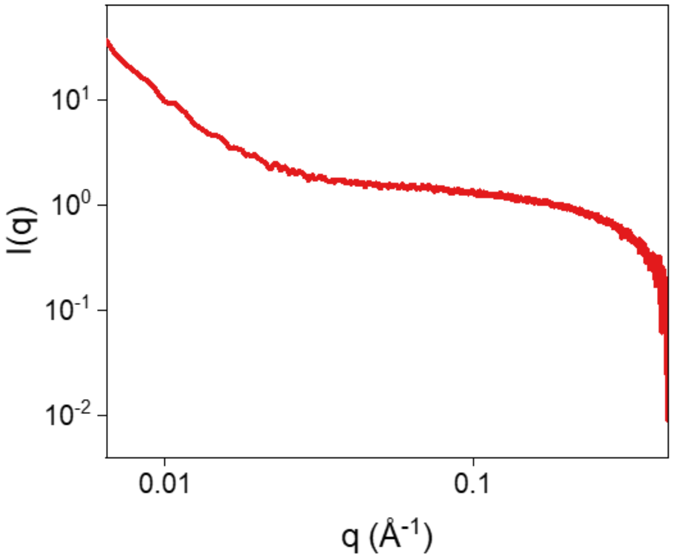

Figure S1. SAXS profiles of lipid mixture.

https://doi.org/10.3390/membranes16060192

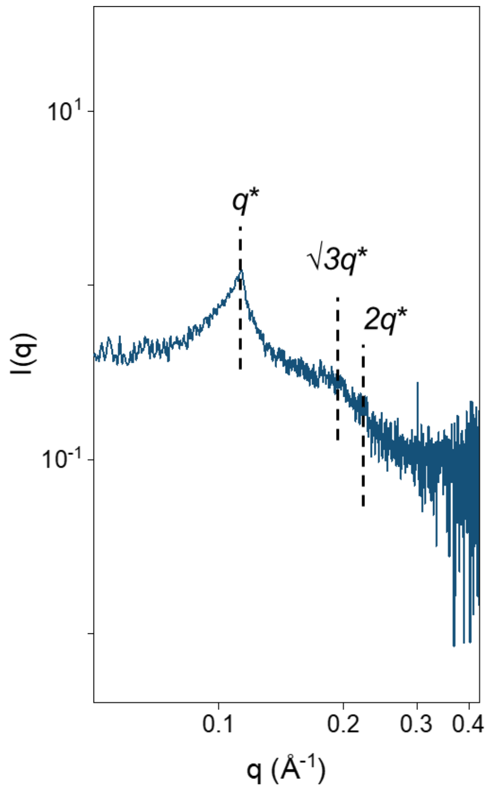

Figure S2. Enlarged view of SAXS profiles of empty-LNPs formulated online at the final stage.

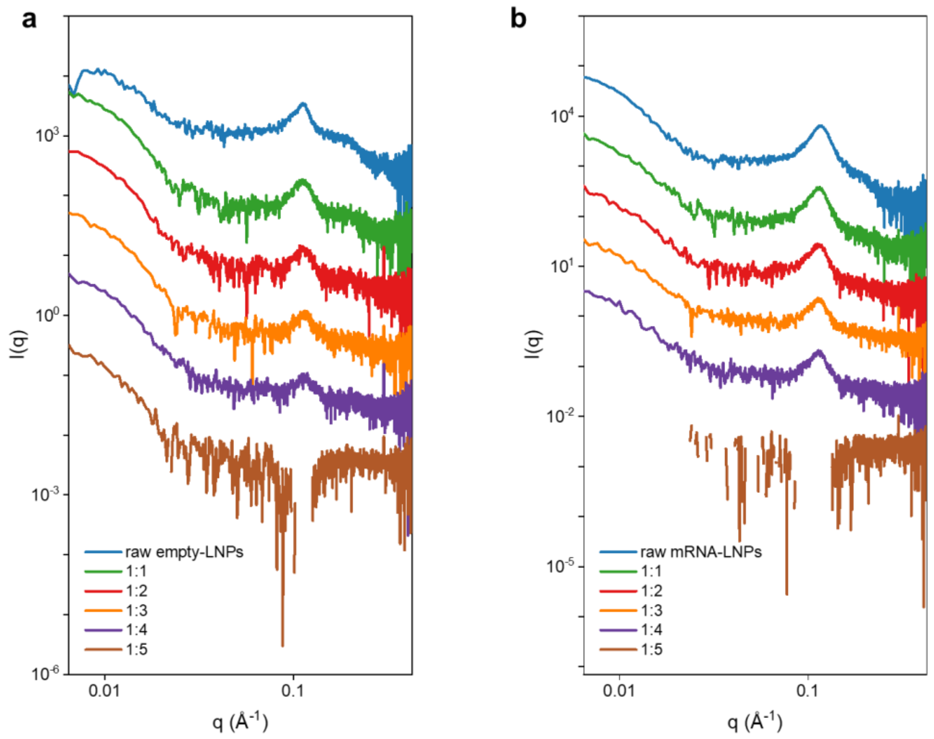

Figure S3. SAXS profiles of (a) empty-LNPs and (b) mRNA-LNPs diluted offline at different ratios. Profiles of 1:5, 1:4, 1:2, 1:1 and raw LNPs are multiplied by a factor of 0.01, 0.1, 10, 100 and 1000, respectively, for better visualization.
